# Investigating the Effect of Line Dipole Magnetic Field on Hydrothermal Characteristics of a Temperature-Sensitive Magnetic Nanofluid Using Two-Phase Simulation

**DOI:** 10.1186/s11671-016-1661-9

**Published:** 2016-10-03

**Authors:** Mehdi Bahiraei, Morteza Hangi

**Affiliations:** 1 Mechanical Engineering Department, School of Energy, Kermanshah University of Technology, Kermanshah, Iran; 2 Research School of Engineering, The Australian National University, Canberra, ACT 2601 Australia

**Keywords:** Magnetic nanofluid, Two-phase simulation, Line dipole, Heat transfer, Parallel plates, Magnetic field

## Abstract

Hydrothermal characteristics of a temperature-sensitive magnetic nanofluid between two parallel plates are investigated in the presence of magnetic field produced by one or multiple line dipole(s) using the two-phase mixture model. As the nanofluid reaches the region where the magnetic field is applied, a rotation is developed due to the dependency of magnetization on temperature. This can lead to mixing in the flow and more uniform distribution of temperature due to the disturbance caused in the boundary layer, and consequently, enhancement in convective heat transfer. The results indicate that the disturbance in boundary layer adjacent to the lower wall is more significant than the upper wall. By application of the magnetic field, the convective heat transfer increases locally for both walls. Due to the intensified mixing, a sudden pressure drop occurs when the fluid reaches the region where the magnetic field is applied. For greater magnetic field strengths and lower Reynolds numbers, the improvement in convective heat transfer is more significant. For small magnetic field strengths, the effect of applying magnetic field on the upper wall is much smaller than that on the lower wall; however, this effect becomes almost the same for both walls at great magnetic field strengths.

## Background

Numerous attempts have been made during the recent years in order to apply different methods to improve heat transfer in various thermal systems. Besides saving a considerable amount of energy, achieving greater rates of heat transfer can lead to the development of more compact heat transfer systems with higher thermal efficiencies. Among different methods, creating secondary flows, flow mixing, flow rotation, and causing disturbance in the boundary layer have been especially taken into account for increasing heat transfer rate in channels. Regarding the fact that further mixing in the flow and, thus, more uniform profiles of temperature and velocity lead to greater heat transfer rate between the flow and the channel walls, application of these methods can be efficient to achieve higher rates of heat transfer. The researchers have utilized various techniques thus far such as different types of baffles [1, 2], vortex generators [3, 4], chaotic geometries [5, 6], and so forth to create mixing in the flow and improve heat transfer.

In addition to the type of the applied geometry, the thermal properties of working fluid are also important in heat transfer processes. Advances in nanotechnology during the recent years have led to the introduction of a new class of suspensions called nanofluids which are obtained from suspension of nanoparticles having proper thermal properties in a base fluid. A great number of studies have examined the effect of using different nanofluids for the improvement of heat transfer rate in various geometries [7–9].

A group of nanofluids, known as magnetic nanofluid or ferrofluid, is a suspension of a base fluid and magnetic nanoparticles covered with a layer of surfactants like oleic acid [10, 11]. Magnetic nanofluids not only demonstrate fluidity like the other fluids but also exhibit magnetic properties similar to magnetic materials. This unique feature makes it possible to control the fluid flow, heat transfer, and the movement of particles by application of an external magnetic field. Thus, magnetic nanofluids can find numerous applications in various fields including bioengineering, electronic packing, and thermal engineering [12–14].

A limited number of studies have been carried out on the flow and heat transfer characteristics of magnetic nanofluids under magnetic fields. Ghofrani et al. [15] performed an experimental investigation to study laminar forced convection of aqueous Fe_3_O_4_ magnetic nanofluid flowing through a circular tube in the presence of an alternating magnetic field. They found that increasing the magnetic field frequency and the nanofluid volume fraction leads to better heat transfer enhancement and the effect of the magnetic field is higher at low Reynolds numbers.

Bahiraei et al. [16] applied the two-phase Euler-Lagrange method to evaluate natural convection of the water-based magnetic nanofluid in a square cavity under nonuniform magnetic field. The applied magnetic field was such that upward magnetic force was applied to the nanoparticles near the hot wall and vice versa near the cold wall. The results indicated that applying the magnetic field can improve the convection of the nanofluid in the cavity and enhance the heat transfer rate.

Aminfar et al. [17] applied a two-phase model to investigate the mixed convection of the water-Fe_3_O_4_ ferrofluid in a vertical rectangular duct under a nonuniform magnetic field generated by an electric current going through a parallel wire located under the duct. They demonstrated that the magnetic field creates a pair of vortices which can improve the heat transfer rate.

Among various magnetic nanofluids, temperature-sensitive magnetic nanofluids show numerous applications in the thermal systems due to their temperature-dependent magnetization. The temperature dependency of magnetization causes the appearance of a nonuniform body force in differentially heated volume of temperature-sensitive magnetic nanofluid, subjected to magnetic field gradients [18]. Therefore, the fluid can be moved under such field-induced thermomagnetic force, and it can be fascinating and promising to employ such an energy conversion process in a range of areas, for instance, thermal management system for some specialized purposes, which is really important for the development of energy systems.

Few studies have been performed on the hydrothermal characteristics of temperature-sensitive magnetic nanofluids. Zablotsky et al. [19] experimentally and numerically investigated thermomagnetic convection of a temperature-sensitive ferrofluid in a rectangular cell in the presence of nonuniform magnetic field provided by permanent magnets attached to the cell walls. They observed that in the presence of magnetic field, enhancement in heat transfer is significantly higher than that in the case of simple thermogravitational convection.

Jin et al. [20] developed a lattice Boltzmann method to simulate the laminar convection of a temperature-sensitive ferrofluid in a porous square cavity under the effect of uniform magnetic field. It was demonstrated that the magnetic force is the main driving force which enhances the average Nusselt number.

Some researchers have focused on the investigation of nanofluids inside parallel-plate channels in the presence of magnetic fields. Malvandi and Ganji [21] assessed the laminar flow and convective heat transfer of nanofluids inside a parallel-plate channel in the presence of a uniform magnetic field. A modified two-component, four-equation, nonhomogeneous equilibrium model was employed, which fully accounted for the effect of the nanoparticle volume fraction distribution. The effects of Brownian and thermophoretic diffusions were considered. The results showed that nanoparticles move from the heated walls towards the core region of the channel and construct a nonuniform nanoparticle distribution. Moreover, in the presence of the magnetic field, the near wall velocity gradients intensified, and therefore, the heat transfer rate and pressure drop increased. Ganguly et al. [22] simulated two-dimensional forced convection heat transfer of a magnetic nanofluid between two parallel plates under the influence of a two-dimensional magnetic field. The magnetic field induced a local vortex near the cold wall. This altered the advection energy transport, changed the temperature distribution in the flow, and enhanced the heat transfer. The authors mentioned that the heat transfer enhancement produced by a magnetic field can be predicted if information regarding the magnetic moment of the field-inducing magnet and the magnetic field distribution are known. Malvandi and Ganji [23] evaluated the effects of nanoparticle migration on mixed convection of nanofluids inside a vertical parallel-plate channel in the presence of a uniform magnetic field. The walls were subjected to different heat fluxes, and nanoparticles were assumed to have a slip velocity relative to the base fluid induced by the Brownian motion and thermophoresis. It was shown that nanoparticles eject themselves from the heated walls, construct a depleted region, and accumulate in the core region, but they were more likely to accumulate towards the wall with the lower heat flux. In addition, inclusion of nanoparticles in the presence of a magnetic field had a negative effect on the performance.

For nanofluids, there are two approaches in numerical simulation: single-phase and two-phase. The single-phase approach in which the effective properties are employed assumes that base fluid and particles are in thermal equilibrium and move with same velocity. However, in two-phase methods, the interaction between the fluid and particles, heat transfer between them and slip velocity are considered. Most numerical studies have applied the single-phase approach, while a limited number of studies have been carried out based on two-phase methods. Some researchers [24, 25] have shown that the two-phase methods give more accurate results in comparison with the single-phase method.

A comprehensive understanding of the relationship between imposed magnetic fields and hydrothermal characteristics of temperature-sensitive magnetic nanofluids is essential for the accurate design and employment of applications involving thermomagnetic convection. The two-phase mixture model is utilized in the current study to numerically analyze effects of the magnetic field produced by one or multiple line dipole(s) on the flow and heat transfer of a temperature-sensitive magnetic nanofluid between two parallel plates. In fact, the magnetic field is applied in order to cause disturbance in the boundary layer and, consequently, enhance the heat transfer rate. The effects of parameters like the magnetic field strength and Reynolds number on temperature, velocity, convective heat transfer, and pressure of the magnetic nanofluid are studied. To our knowledge, this survey is the first study that employs a two-phase simulation to examine the hydrothermal characteristics of a temperature-sensitive ferrofluid under the influence of a line dipole.

## Methods

### Definition of Geometry and Applied Magnetic Field

**Fig. 1:**
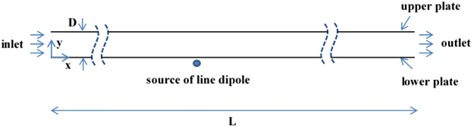
Geometry under study

The magnetic nanofluid flows between two parallel flat plates. The length of the plates is *L*, and the distance between them is *D*. The origin of the Cartesian coordinate is located at the leading edge of the lower plate (see Fig. 1). As can be seen in Fig. 1, a line dipole which provides the external magnetic field is located under the lower plate at *x*/*D* = 50 and *y*/*D* = −0.1. The resulting field is two-dimensional which can be expressed in polar coordinate as below [26]:

1 $$ \mathbf{H}\left(r,\theta \right)=m\left(\frac{ \sin \theta }{r^2}{\widehat{e}}_r-\frac{ \cos \theta }{r^2}{\widehat{e}}_{\theta}\right) $$

where **H** is the vector of the magnetic field strength, *m* denotes the magnetic dipole moment which determines the strength of the dipole, and *r* and *θ* are respectively the radial and peripheral directions of polar coordinate in which *ê*_*r*_ and *ê*_*θ*_ are unit vectors. It should be noted that this correlation is for the case that the origin of the polar coordinate is placed at the source of the dipole. Therefore, in order to use the above magnetic field correlation, it is converted into the Cartesian coordinate with its origin being mentioned above.

The working fluid is a temperature-sensitive magnetic nanofluid; the properties of which are summarized in Table 1. It consists of Mn-Zn ferrite nanoparticles suspended in tetradecane.

**Table 1:** Properties of the magnetic nanofluid [19]

Property	Value
Density	976 kgm^−3^
Dynamic viscosity	0.006 Pas
Specific heat	2190 Jkg^−1^K^−1^
Thermal conductivity	0.14 Wm^−1^K^−1^
Particle diameter	~10 nm
Volumetric concentration	6 %
Pyromagnetic coefficient	0.0028 K^−1^

### Mathematical Formulation

The two-phase mixture model is employed to simulate the hydrothermal characteristics of the temperature-sensitive ferrofluid flowing between two parallel plates under the effect of magnetic field produced by one or multiple line-source dipole(s). In this method, it is supposed that the coupling between the phases is strong. Also, the phases have their own velocity fields, and the relative velocity between the phases is taken into account. Considering these assumptions, the conservation equations are written as below [27–29]:

Continuity equation:

2 $$ \nabla .\left({\rho}_m{\mathrm{v}}_m\right)=0 $$

Momentum equation:

3 $$ \nabla .\left({\rho}_m{\mathrm{v}}_m{\mathrm{v}}_m\right)=-\nabla P+\nabla .\left({\mu}_m\nabla {\mathrm{v}}_m\right)+\nabla .\left({\varphi}_p{\rho}_p{\mathrm{v}}_{dr,p}{\mathrm{v}}_{dr,p}\right)+{\mu}_0M\nabla H+{\mu}_0H\frac{\partial M}{\partial T}\nabla T $$

Energy equation:

4 $$ \nabla .\left(\left({\varphi}_p{\rho}_p{c}_{p,p}{\mathrm{v}}_p+\left(1-{\varphi}_p\right){\rho}_f{c}_{p,f}{\mathrm{v}}_f\right)T\right)=\nabla .\left({k}_m\nabla T\right) $$

Volume fraction:

5 $$ \nabla .\left({\varphi}_p{\rho}_p{\mathrm{v}}_m\right)=-\nabla .\left({\varphi}_p{\rho}_p{\mathrm{v}}_{dr,p}\right) $$

where

6 $$ {\mathrm{v}}_m=\frac{\varphi_p{\rho}_p{\mathrm{v}}_p+\left(1-{\varphi}_p\right){\rho}_f{\mathrm{v}}_f}{\rho_m} $$

In Eqs. 2, 3, 4, 5, and 6, subscripts *m*, *p*, and *f* refer to mixture, particle, and base fluid, respectively. Furthermore, *ρ*, **v**, *μ*, φ, *c*_*p*_, *k*, *P*, and *T* represent density, velocity, dynamic viscosity, volume concentration, specific heat, thermal conductivity, pressure, and temperature, respectively. In addition, *μ*_0_ and *M* denote the permeability of vacuum and magnetization of the ferrofluid, respectively.

The last two terms on the right side of Eq. 3 describe the magnetic forces applied to the ferrofluid which are respectively induced by the nonuniform magnetic field and the temperature gradient [30]. For the temperature-sensitive ferrofluid exposed to a nonuniform magnetic field, both of these two terms are effective on the fluid flow. In the mentioned terms, the magnetization of the ferrofluid which is dependent on temperature is evaluated as below [19]:

7 $$ M(T)={M}_{\mathrm{ref}}\left(1-{\beta}_m\left(T-{T}_{\mathrm{ref}}\right)\right) $$

where *β*_*m*_ represents the pyromagnetic coefficient of the ferrofluid which indicates the dependency of the magnetization on temperature. Additionally, *M*_ref_ which is the magnetization at reference temperature (*T*_ref_) can be obtained according to the single-parameter Langevin approximation as below:

8 $$ {M}_{ref}=\varphi {M}_sL\left(\zeta \right) $$

where *M*_*s*_ denotes the saturation magnetization of magnetic particles and *ζ* is the argument of Langevin function (*L*) as below:

9 $$ \zeta =\frac{\mu_0{m}_pH}{k_BT} $$

where *k*_*B*_ represents Boltzmann constant and *m*_*p*_ is magnetic moment of nanoparticle.

The Langevin function is defined as follows:

10 $$ L\left(\zeta \right)= \coth \zeta -\frac{1}{\zeta } $$

In Eq. 3, **v**_*dr*,*p*_ is the drift velocity for the secondary phase (i.e., nanoparticles) and is defined as below:

11 $$ {\mathrm{v}}_{dr,p}={\mathrm{v}}_p-{\mathrm{v}}_m $$

The slip velocity (i.e., relative velocity) is the velocity of the secondary phase (*p*) relative to the velocity of the primary phase (*f*):

12 $$ {\mathrm{v}}_{pf}={\mathrm{v}}_p-{\mathrm{v}}_f $$

The relation between the drift velocity and the relative velocity is as below:

13 $$ {\mathrm{v}}_{dr,p}={\mathrm{v}}_{pf}-\frac{\varphi_p{\rho}_p}{\rho_m}\left({\mathrm{v}}_f-{\mathrm{v}}_p\right) $$

Considering the forces acting on a single magnetic particle, the slip velocity is obtained similar to Jafari et al. [31] as follows:

14 $$ {\mathrm{v}}_{pf}=\frac{C_c{\mu}_0{m}_pL\left(\zeta \right)}{3\pi {\mu}_f{d}_p}\nabla H+\frac{\rho_p{d}_p^2{C}_c}{18{\mu}_f}\frac{\rho_p-{\rho}_f}{\rho_p}\mathbf{g} $$

It should be noted that in the above equation, a form of Stokes’ drag law for sub-micron particles [32] has been employed, where *d*_*p*_ represents the nanoparticle diameter and **g** is gravitational acceleration. Furthermore, *C*_*c*_ is the Cunningham correction factor to Stokes’ drag law which can be evaluated from:

15 $$ {C}_c=1+\frac{2\lambda }{d_p}\left(1.257+0.4{e}^{-\left(1.1{d}_p/2\lambda \right)}\right) $$

where *λ* denotes molecular mean free path for the base fluid.

### Boundary Conditions

Uniform velocity is considered at the entrance, and therefore, the hydrodynamic boundary layer is developing. Moreover, the problem is steady, and no-slip condition at the walls and zero relative pressure at the outlet are employed. Uniform temperature is utilized at the channel inlet, and equal and constant heat fluxes are applied at upper and lower plates from the inlet of the channel. Hence, the problem is developing thermally.

### Numerical Method and Validation

The equations were discretized applying control volume technique. QUICK method was used to solve the convection term, while SIMPLE method was adopted for velocity-pressure coupling. Grids with different meshes were examined to ensure the grid independency, and finer meshing was implemented near the walls due to the presence of severe temperature and velocity gradients.

**Fig. 2:**
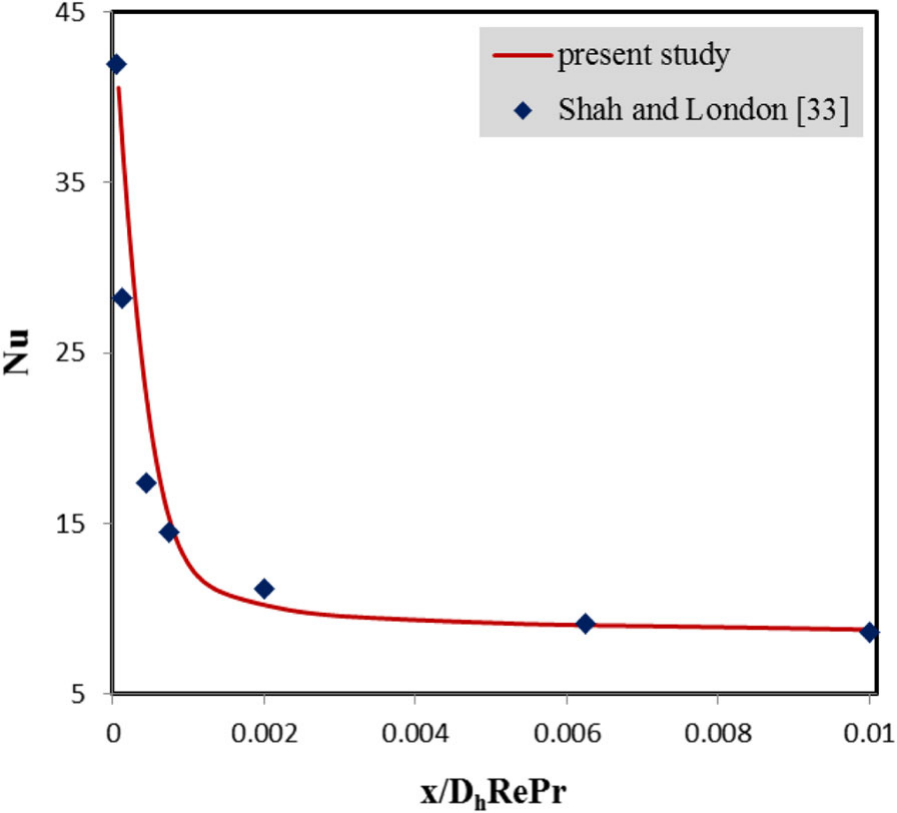
Nusselt number obtained from present simulations compared to the one presented by Shah and London [33]

To validate the numerical method, Nusselt number obtained from the present simulations was compared with the one presented by Shah and London [33] for pure water flowing between two parallel plates. As seen in Fig. 2, there is a good agreement between the results. In addition, the convective heat transfer coefficient obtained from the current simulation method was compared with the one obtained from the experimental study of Wen and Ding [34] for water-Al_2_O_3_ nanofluid flow inside a horizontal tube at φ = 1 % and Re = 1600. The results are presented in Table 2, and as can be observed, there is a proper consistency between the results showing the validity of the numerical method.

**Table 2:** Results obtained from current study in comparison with the experimental results [34] for water-Al_2_O_3_ nanofluid at φ = 1 % and Re = 1600

*h* (W/m^2^K)			
Dimensionless axial distance	Wen and Ding [34]	Present study	Error (%)
26.2	2611.5	2697.7	3.3
62.8	1741.6	1692.4	2.8
115.7	1201.1	1221.1	1.7
146.1	1015.2	993.5	2.1
172.3	905.4	887.9	1.9

## Results and Discussion

Hydrothermal characteristics of the magnetic nanofluid flow between the two parallel plates under the magnetic field produced by one or multiple line dipole(s) are studied using the two-phase mixture method. First, the effect of applying the magnetic field of one line dipole on temperature, velocity, convective heat transfer, and pressure changes of the ferrofluid is assessed at various Reynolds numbers and magnetic field strengths. Then, the magnetic fields are applied in different regions with their effects being evaluated.

**Fig. 3:**
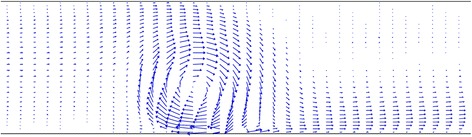
Velocity vectors of the magnetic nanofluid at Re = 500 and *m* = 0.05 Am

**Fig. 4:**
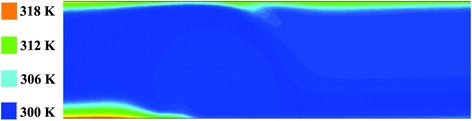
Temperature contour at Re = 500 and *m* = 0.05 Am

Figure 3 depicts velocity vectors of the magnetic nanofluid close to the region where the magnetic field is applied for *m* = 0.05 Am and Re = 500. As can be observed, when the fluid approaches this region, a rotation is developed in the flow. This observation can be attributed to the fact that the magnetization is dependent on the temperature in the temperature-sensitive ferrofluid, such that it decreases at higher temperatures. This dependency causes the fluid flowing upstream the region of the applied magnetic field, and the fluid flowing in central regions, which are colder than the fluid adjacent to the lower plate, to be attracted more towards the magnetic field source, and causes the hot fluid to be repelled from the lower plate. In other words, the colder magnetic fluid experiences a greater magnetic force as compared to the hot one. Therefore, the application of such magnetic field causes mixing in the flow, and as seen in Fig. 4, in which the temperature contour is depicted, the colder fluid comes closer to the lower hot plate, which can lead to increasing the heat transfer rate.

Another point which can be noticed in Fig. 4 is that before the fluid reaches the region where the magnetic field is applied, the thermal boundary layer is growing. However, once the fluid reaches this region and by development of rotation in the flow, as discussed above, a sudden disturbance is created at the boundary layer and its growth is stopped. When the fluid passes by this region, the thermal boundary layer begins to grow again. Moreover, it is observed that the disturbance in the thermal boundary layer is more intensive adjacent to the lower wall in comparison with the upper wall, since according to Fig. 1, the source of the magnetic field is located below the lower plate, and consequently, the magnetic field gradient is more significant there.

**Fig. 5:**
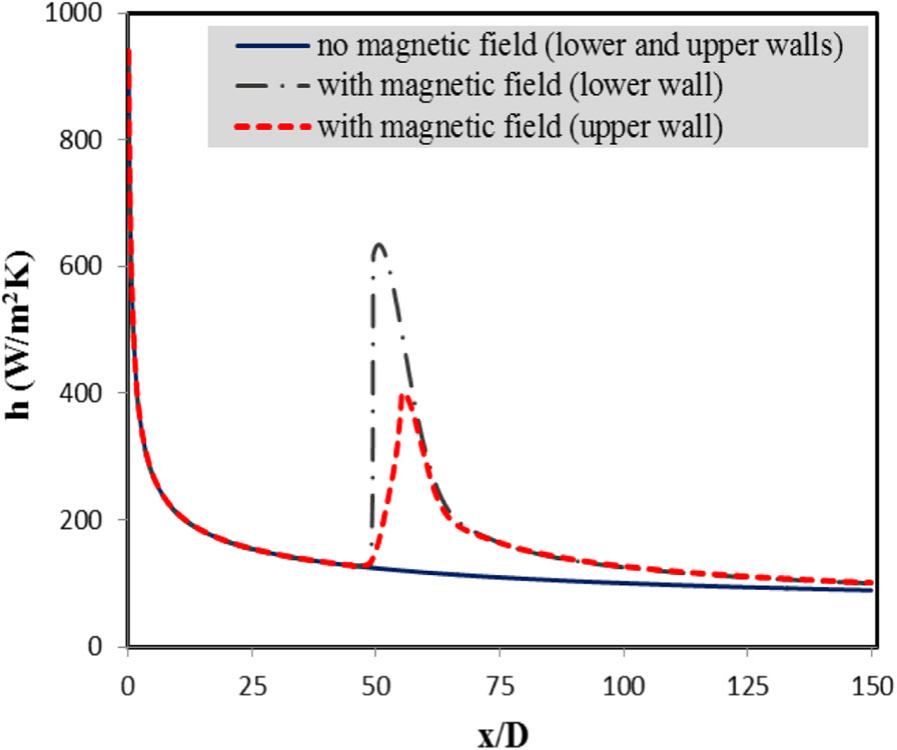
Convective heat transfer coefficients at the upper and lower walls in the presence of the magnetic field for Re = 500 and *m* = 0.05 Am compared to those for the case without magnetic field

In Fig. 5, the convective heat transfer coefficients at the upper and lower walls in the presence of the magnetic field for Re = 500 and *m* = 0.05 Am are compared with those for the case without magnetic field. As seen from this figure, without the magnetic field, the convective heat transfer coefficients are exactly the same at the upper and lower walls, since there is no mixing in flow and also similar heat flux is applied to them. It is also observed that where the magnetic field is applied, the convective heat transfer coefficients at both walls increase locally. The reason is that, as shown in Fig. 4, in the region of applying the magnetic field, the temperature is distributed more uniformly in the cross section due to the mixing developed, especially near the lower wall. According to the equation of the convective heat transfer coefficient (i.e., *h* = *q*"/(*T*_*s*_ − *T*_*b*_)), the more uniform the temperature distribution, the lower the difference between the wall temperature (*T*_*s*_) and the bulk temperature of the fluid (*T*_*b*_), and therefore, the higher the convective heat transfer coefficient. It is additionally noticed that with application of the magnetic field, the convective heat transfer increment is more significant in the lower wall. Meanwhile, as the magnetic field and its gradient decrease quickly once the fluid passes by the dipole, the effect of the magnetic field on the flow is strictly local, and the convective heat transfer coefficient decreases again along the *x*-axis once the fluid passes by this region, due to the growth of the thermal boundary layer.

**Fig. 6:**
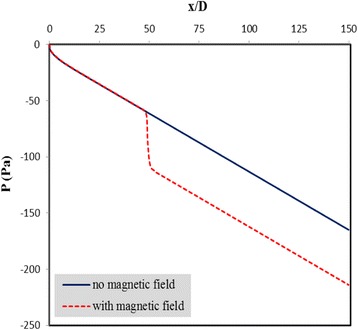
Variations of the relative pressure of the nanofluid along the channel length for cases with and without magnetic field at *m* = 0.05 Am and Re = 500

Variations of the relative pressure of the nanofluid along the channel length are shown in Fig. 6 for cases with and without the magnetic field at *m* = 0.05 Am and Re = 500. When the fluid reaches the region where the magnetic field is applied, there is a sudden pressure drop which is attributed to the mixing developed within the flow and the disturbance of the hydrodynamic boundary layer. Once the fluid passes by this region, the hydrodynamic boundary layer begins to grow again such that the trend of the pressure change in the regions after the dipole source is similar to its trend before the fluid reaches this region.

**Fig. 7:**
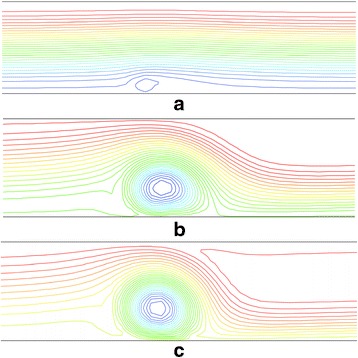
Streamlines in the regions of the applied magnetic field at Re = 1000 for the magnetic dipole moment of (**a**) 0.01 Am, **b** 0.05 Am, and **c** 0.1 Am

Streamlines are illustrated in Fig. 7 in the regions of the applied magnetic field at Re = 1000 for the magnetic dipole moment of 0.01, 0.05, and 0.1 Am. As can be observed, the stronger the magnetic field, the greater the flow rotation. The reason is that at higher values of *m*, larger area of the channel will be affected by the magnetic field and also the magnetic field gradient becomes more significant. Consequently, the fluid flow will experience a greater magnetic force, and the flow mixing will increase.

**Fig. 8:**
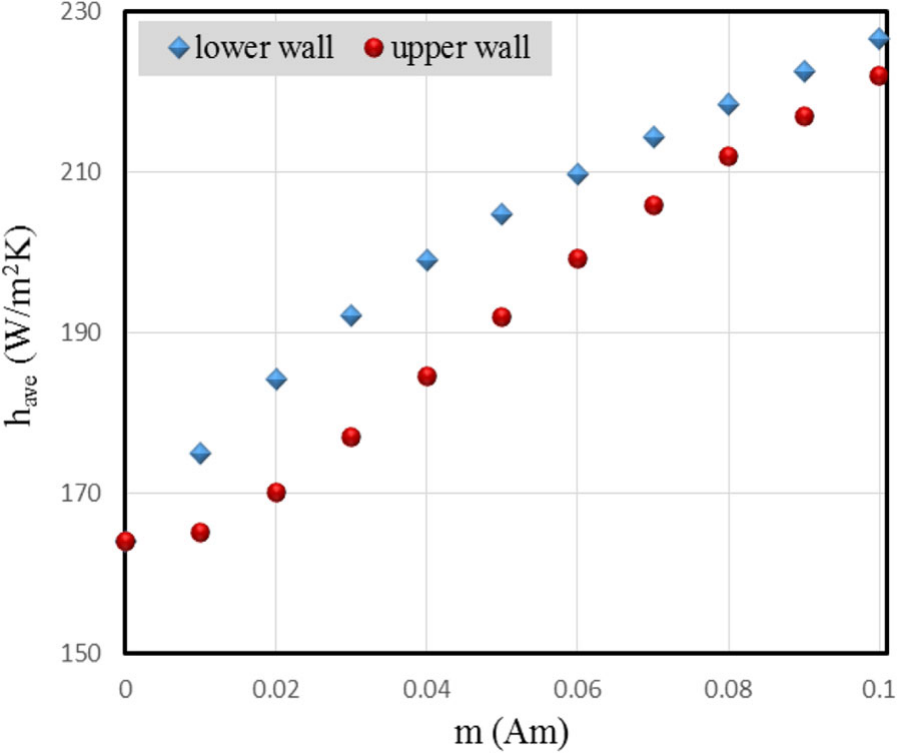
Average convective heat transfer coefficients at the upper and lower walls in terms of the magnetic dipole moment at Re = 1000

The average convective heat transfer coefficients at the upper and lower walls are depicted in Fig. 8 in terms of the magnetic dipole moment at Re = 1000. It can be seen that the average convective heat transfer coefficient increases by increasing *m*. The other point which can be observed is that at smaller values of *m*, the effect of the magnetic field on the upper wall is much smaller than that on the lower wall; however, this effect becomes almost the same for both walls at greater values of *m*. The reason is that, as seen in Fig. 7, increasing the magnetic dipole moment intensifies the rotation developed in the flow, and thus, effectiveness of the magnetic field increases on the upper wall. Moreover, it is clear from Fig. 8 that the change of *h*_ave_ in terms of *m* for the upper wall is almost linear and for the lower wall is parabolic. By curve fitting, the relations between *h*_ave_ and *m* for the upper and lower walls are obtained as below:

Upper wall:

16 $$ {h}_{\mathrm{ave}}=631.96m+160.12 $$

Lower wall:

17 $$ {h}_{\mathrm{ave}}=-3767.1{m}^2+978.56m+165.22 $$

**Fig. 9:**
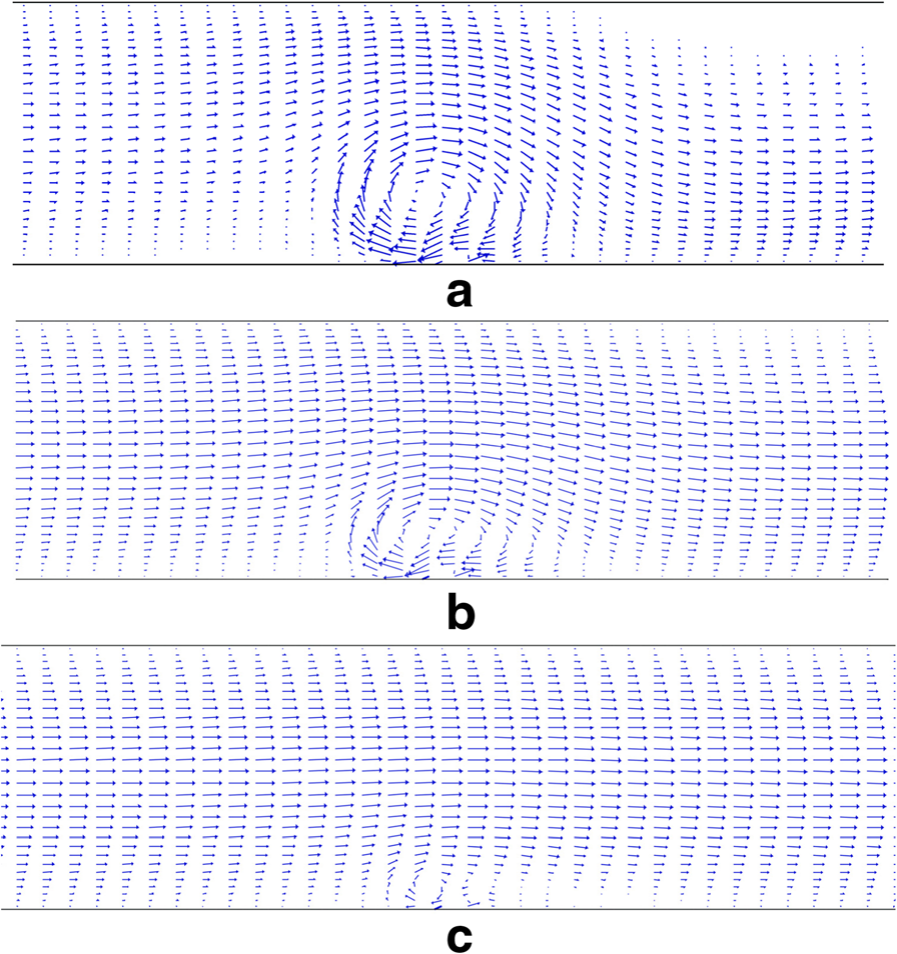
Velocity vectors of the nanofluid for *m* = 0.01 Am at (**a**) Re = 500, **b** Re = 1000, and **c** Re = 1500

In Fig. 9, the velocity vectors are shown at different Reynolds numbers for *m* = 0.01 Am. It is noteworthy that the Reynolds number is defined by Eq. 18. As can be noticed in this equation, the mixture properties have been employed for definition of the Reynolds number. It means that the Reynolds number is dependent on the nanofluid properties; however, only one value for the nanofluid concentration (i.e., φ = 6 %) has been considered in the present study.

18 $$ \mathrm{R}\mathrm{e}=\frac{\rho_mvD}{\mu_m} $$

At higher Reynolds numbers, the effect of the magnetic field on the flow reduces due to greater inertia of the flow, and as can be observed, the rotation developed in the flow becomes smaller. As a result, by applying the magnetic field at higher Reynolds numbers, smaller growth will be noticed in the convective heat transfer, such that for Re = 1000, the enhancement in the convective heat transfer coefficient of the lower wall under the magnetic field will be approximately 6.6 % in comparison with the case without magnetic field, while this value is 17.9 % for Re = 500.

**Fig. 10:**
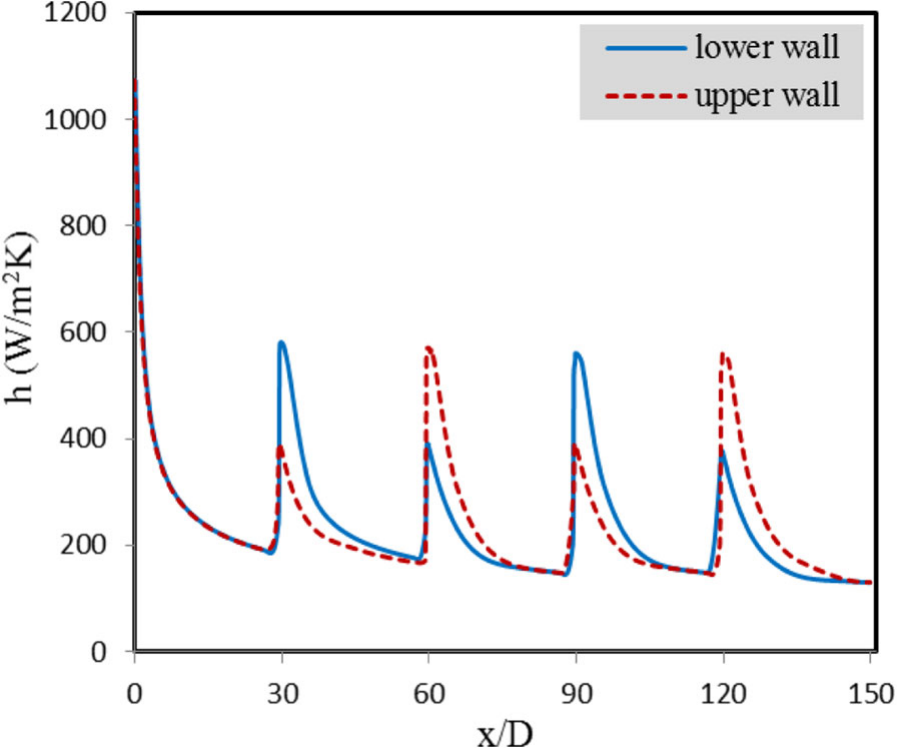
Convective heat transfer coefficients at both walls for Re = 1000 and *m* = 0.05 Am

As was observed, using such a magnetic field in a section of the channel will lead to disturbance of the boundary layer, development of mixing in the flow, and local improvement of the heat transfer rate. The effect of using four line dipoles (two below the channel and two above the channel) is now studied. For this case, the convective heat transfer coefficients at both walls are illustrated in Fig. 10 for Re = 1000 and *m* = 0.05 Am. The positions of the dipoles with respect to the origin of the Cartesian coordinate, depicted in Fig. 1, are presented below:

Dipole 1: *x*/*D* = 30 and *y*/*D* = −0.1

Dipole 2: *x*/*D* = 60 and *y*/*D* = 1.1

Dipole 3: *x*/*D* = 90 and *y*/*D* = −0.1

Dipole 4: *x*/*D* = 120 and *y*/*D* = 1.1

As can be observed, where the magnetic field is applied, the amount of convective heat transfer increases at both walls, such that for the sections where dipoles are close to the lower wall (i.e., dipoles 1 and 3), the intensity of this enhancement in the lower wall is more significant than that in the upper wall. However, for the sections where dipoles are close to the upper wall (i.e., dipoles 2 and 4), the increase in the convective heat transfer is greater at the upper wall. Generally speaking, application of these magnetic fields has caused the average heat transfer coefficient to be increased by about 50 % at both walls.

It can be noted that induction of such mixing in the flow can not only increase the heat transfer but also avoid sedimentation of the nanoparticles. Assessing different arrangements of the line dipoles is important since a realistic magnetic field formed by a permanent magnet or an electromagnet having different sizes can be produced by a number of properly placed line dipoles.

Magnetic nanofluids have promising potential for thermal applications, because their convection can be easily controlled by applying an external magnetic field. However, unlike conventional forced and free convections, this type of convection is not yet properly characterized. Although the hydrothermal characteristics of a magnetic nanofluid under the effect of the magnetic field produced by line dipoles were evaluated in the current study, much more investigations are necessary both experimentally and numerically in order to better identify the characteristics of this class of nanofluids. In future studies, it is suggested to pay more attention to the two-phase methods in order to gain better understanding of the ferrofluid behavior in the presence of magnetic fields.

## Conclusions

In this numerical study, the flow of a temperature-sensitive magnetic nanofluid between two parallel plates was assessed under the effect of the magnetic field produced by one or multiple line dipole(s). Applying the magnetic field led to greater local values of the convective heat transfer at both upper and lower walls. Since the magnetic field source is located adjacent to the lower wall, the disturbance of the boundary layer adjacent to this wall was more significant, and thus, the heat transfer augmented more in this region. For greater magnetic field strengths and lower Reynolds numbers, the fluid rotation was stronger and the enhancement in the convective heat transfer was greater. For example at Re = 1000, the amount of enhancement in the convective heat transfer coefficient of the lower wall was about 6.6 % in comparison with the case without magnetic field. However, this amount was reported to be 17.9 % for Re = 500. For small magnetic field strengths, the effect of the magnetic field on the upper wall was much smaller than that on the lower wall, though this effect on the upper wall became more significant by increasing the magnetic field strength due to greater rotation caused in the flow. Furthermore, disturbance of the hydrodynamic boundary layer due to application of the magnetic field led to greater pressure drop in the nanofluid flow. Finally, since various arrangements of line dipoles can simulate practical magnetic fields, the effect of using four line dipoles on the convective heat transfer was evaluated, and this arrangement caused about 50 % improvement in the average convective heat transfer coefficients at both walls.

## Nomenclature

*C*_*c*_ Cunningham correction factor

*C*_*p*_ specific heat, J/kgK

*d*_*p*_ diameter of nanoparticle, m

***g*** gravitational acceleration

*H* magnetic field strength

*h* convective heat transfer coefficient, W/m^2^K

_*k*_ thermal conductivity,W/mK

*k*_*B*_ Boltzman constant, J/K

*M* magnetization

*M*_*s*_ saturation magnetization

*m* magnetic dipole moment

*L* Langevin function

_*P*_ pressure, Pa

_*T*_ temperature, K

V velocity, m/s

_*vdr*,*p*_ drift velocity, m/s

_*vpf*_ relative velocity, m/s

*Greek symbols*

*β* pyromagnetic coefficient

*ζ* argument of Langevin function

_*λ*_ mean free path, m

*μ* dynamic viscosity, Pa.s

*μ*_*0*_ permeability of vacuum

*ρ* density, Kg/m^3^

φ volume concentration

*Subscripts*

*f* base fluid

*m* mixture

*p* particle

## References

[1] Elias MM, Shahrul IM, Mahbubul IM, Saidur R, Rahim NA (2014). Effect of different nanoparticle shapes on shell and tube heat exchanger using different baffle angles and operated with nanofluid. Int J Heat Mass Transf.

[2] Bahiraei M, Hangi M, Saeedan M (2015). A novel application for energy efficiency improvement using nanofluid in shell and tube heat exchanger equipped with helical baffles. Energy.

[3] Xia HH, Tang GH, Shi Y, Tao WQ (2014). Simulation of heat transfer enhancement by longitudinal vortex generators in dimple heat exchangers. Energy.

[4] Khoshvaght-Aliabadi M (2016) Thermal performance of plate-fin heat exchanger using passive techniques: vortex-generator and nanofluids. Heat Mass Transf 52:819–28

[5] Zheng Z, Fletcher DF, Haynes BS (2013). Chaotic advection in steady laminar heat transfer simulations: periodic zigzag channels with square cross-sections. Int J Heat Mass Transf.

[6] Bahiraei M, Hangi M (2016). Numerical investigation and optimization of flow and thermal characteristics of nanofluid within a chaotic geometry. Adv Powder Technol.

[7] Lotfi R, Rashidi AM, Amrollahi A (2012). Experimental study on the heat transfer enhancement of MWNT-water nanofluid in a shell and tube heat exchanger. Int Communic Heat Mass Transf.

[8] Kannadasan N, Ramanathan K, Suresh S (2012). Comparison of heat transfer and pressure drop in horizontal and vertical helically coiled heat exchanger with CuO/water based nanofluids. Exp Therm Fluid Sci.

[9] Bahiraei M (2016). Particle migration in nanofluids: a critical review. Int J Therm Sci.

[10] Odenbach S (2002). Ferrofluids.

[11] Rosensweig RE (1985). Ferrohydrodynamics.

[12] Miaskowski A, Sawicki B (2013). Magnetic fluid hyperthermia modeling based on phantom measurements and realistic breast model. IEEE Transac Biomedical Eng.

[13] Lee YW, Chang TL (2013). Novel perturbations between magnetic nanofluid and the thermal fluidic system at heat dissipation. Microelectronic Eng.

[14] Bahiraei M, Hangi M (2015). Flow and heat transfer characteristics of magnetic nanofluids: a review. J Magn Magn Mater.

[15] Ghofrani A, Dibaei MH, Hakim Sima A, Shafii MB (2013). Experimental investigation on laminar forced convection heat transfer of ferrofluids under an alternating magnetic field. Exp Therm Fluid Sci.

[16] Bahiraei M, Hangi M (2014). Natural convection of magnetic nanofluid in a cavity under non-uniform magnetic field: a novel application. J Supercond Nov Magn.

[17] Aminfar H, Mohammadpourfard M, Ahangar Zonouzi S (2013). Numerical study of the ferrofluid flow and heat transfer through a rectangular duct in the presence of a non-uniform transverse magnetic field. J Mag Mag Mater.

[18] Blums E, Cebers A, Maiorov MM (1997). Magnetic fluids.

[19] Zablotsky D, Mezulis A, Blums E (2009). Surface cooling based on the thermomagnetic convection: numerical simulation and experiment. Int J Heat Mass Transf.

[20] Jin L, Zhang XR (2013). Analysis of temperature-sensitive magnetic fluids in a porous square cavity depending on different porosity and Darcy number. Appl Therm Eng.

[21] Malvandi A, Ganji DD (2014). Magnetic field effect on nanoparticles migration and heat transfer of water/alumina nanofluid in a channel. J Magn Magn Mater.

[22] Ganguly R, Sen S, Puri IK (2004). Heat transfer augmentation using a magnetic fluid under the influence of a line dipole. J Magn Magn Mater.

[23] Malvandi A, Ganji DD (2015). Effects of nanoparticle migration on hydromagnetic mixed convection of alumina/water nanofluid in vertical channels with asymmetric heating. Phys E.

[24] Haghshenas Fard M, Esfahany MN, Talaie MR (2010). Numerical study of convective heat transfer of nanofluids in a circular tube two-phase model versus single-phase model. Int Communic Heat Mass Transf.

[25] Bahremand H, Abbassi A, Saffar-Avval M (2015). Experimental and numerical investigation of turbulent nanofluid flow in helically coiled tubes under constant wall heat flux using Eulerian–Lagrangian approach. Powder Technol.

[26] Ganguly R, Sen S, Puri IK (2004). Thermomagnetic convection in a square enclosure using a line dipole. Phys Fluids.

[27] Mirmasoumi S, Behzadmehr A (2012). Effects of nanoparticles mean diameter on the particle migration and thermo-hydraulic behavior of laminar mixed convection of a nanofluid in an inclined tube. Heat Mass Transf.

[28] Aminfar H, Mohammadpourfard M, Narmani Kahnamouei Y (2011). A 3D numerical simulation of mixed convection of a magnetic nanofluids in the presence of non-uniform magnetic field in a vertical tube using two phase mixture model. J Magn Magn Mater.

[29] Bahiraei M, Hangi M (2016). Automatic cooling by means of thermomagnetic phenomenon of magnetic nanofluid in a toroidal loop. Appl Therm Eng.

[30] Lian W, Xuan Y, Li Q (2009). Characterization of miniature automatic energy transport devices based on the thermomagnetic effect. Energ Convers Manage.

[31] Jafari A, Tynjala T, Mousavi SM, Sarkomaa P (2008). Simulation of heat transfer in a ferrofluid using computational fluid dynamics technique. Int J Heat Fluid Flow.

[32] Ounis H, Ahmadi G, McLaughlin JB (1991). Brownian diffusion of submicrometer particles in the viscous sublayer. J Colloid Interface Sci.

[33] Shah RK, London AL (1978). Laminar flow forced convection in ducts.

[34] Wen D, Ding Y (2004). Experimental investigation into convective heat transfer of nanofluids at the entrance region under laminar flow conditions. Int J Heat Mass Transf.

